# Does the millennial generation of women experience more mental illness than their mothers?

**DOI:** 10.1186/s12888-021-03361-5

**Published:** 2021-07-17

**Authors:** Jake M. Najman, William Bor, Gail M. Williams, Christel M. Middeldorp, Abdullah A. Mamun, Alexandra M. Clavarino, James G. Scott

**Affiliations:** 1 School of Public Health, Public Health Building, The University of Queensland, Herston, 4006 Australia; 2 Mater Child and Youth Mental Health Service, Mater Hospital, The University of Queensland, South Brisbane, Australia; 3 Child Health Research Centre, The University of Queensland, South Brisbane, Australia; 4 Child and Youth Mental Health Service, Children’s Health Queensland Hospital and Health Service, South Brisbane, Australia; 5 Institute for Social Science Research, The University of Queensland, Indooroopilly, Australia; 6 Mental Health Research Programme, QIMR Berghofer Medical Research Institute, Herston, Australia; 7 Metro North Mental Health Service, Herston, Qld Australia

**Keywords:** Depression, Anxiety, Post-traumatic stress disorder, Generational changes, Prevalence

## Abstract

**Background:**

There is concern that rates of mental disorders may be increasing although findings disagree. Using an innovative design with a daughter-mother data set we assess whether there has been a generational increase in lifetime ever rates of major depressive disorder, generalised anxiety disorder, panic disorder, and post-traumatic stress disorder (PTSD) experienced prior to 30 years of age.

**Methods:**

Pregnant women were recruited during 1981–1983 and administered the Composite International Diagnostic Interview (CIDI) at the 27-year follow-up (2008–11). Offspring were administered the CIDI at the 30-year follow-up (2010–2014). Comparisons for onset of diagnosis are restricted to daughter and mother dyads up to 30 years of age. To address recall bias, disorders were stratified into more (≥12 months duration) and less persistent episodes (< 12 months duration) for the purposes of comparison. Sensitivity analyses with inflation were used to account for possible maternal failure to differentially recall past episodes.

**Results:**

When comparing life time ever diagnoses before 30 years, daughters had higher rates of persistent generalised anxiety disorder, and less persistent major depressive disorder, generalised anxiety disorder and PTSD.

**Conclusions:**

In the context of conflicting findings concerning generational changes in mental disorders we find an increase in generational rates of persistent generalised anxiety disorders and a range of less persistent disorders. It is not clear whether this finding reflects actual changes in symptom levels over a generation or whether there has been a generational change in recognition of and willingness to report symptoms of mental illness.

**Supplementary Information:**

The online version contains supplementary material available at 10.1186/s12888-021-03361-5.

## Background

In the context of societies experiencing rapid social change, there are concerns that the mental health of more recent generations is declining. There is ample evidence of recent social and structural changes in behaviour and the lifestyles of younger generations. These changes are in four broad categories and encompass almost every aspect of life. Firstly, there have been fundamental demographic changes in family and work life that could have a negative impact on mental health [1–3]. Societies are more urbanised than ever before, with higher density living, reduced opportunities for physical activity and increases in obesity [4]. Secondly, it is suggested there has been a shift from communal aspirations towards individual goals emphasising wealth, appearance, status and personal attainment [5, 6]. There is some evidence that younger generations are more narcissistic than previous generations – a shift to generation “me” from generation “we” [3]. Thirdly, more recent generations live in a media environment which dominates their day-to-day activities. More recent generations are more (electronically) connected, but possibly more physically isolated from social networks than ever before [7]. Fourthly, young people are delaying attaining what were previously important developmental milestones such as employment, marriage, having children and buying a home [8, 9].

Arguably these four types of societal changes could negatively influence mental health at a population level. However, it is not at all clear that current generations are experiencing more rapid or substantial change than did previous generations, nor that these changes lead to increased rates of mental illness. Arguably, all generations experience what are for that generation, major social change.

In the context of ongoing rapid social change, some suggest there have been generational increases in rates of mental illness [6, 10–13]. A meta-analysis of 131 studies has suggested that there may have been increases in mental illness over time in North America but not in some European populations [14]. The above findings contrast with a body of research suggesting an absence of change in psychopathology over time or across generations. A systematic review of studies using similar measures of mental illness pointed to inconsistent results but with the possibility that there had been an increase in internalizing symptoms experienced by young females [15]. A meta-analysis of 26 studies, restricted to using concurrently administered measures of depression, found no change over time for child or adolescent psychopathology [16]. Other studies have found no changes [17] or no consistent changes over time in the prevalence of depressive symptoms or disorders [4].

The conflicting findings should be interpreted in context. There is no doubt that in economically advanced countries, there has been a substantial increase in the availability and use of treatment services for those with mental illness [18–20]. It is unclear whether this increase is related to the incidence and/or prevalence of mental illness in a population or rather a consequence of increased level of service provision and/or mental health literacy in high income countries [21]. Much of the available data which is used to test the case for changes in mental illness over time involve comparisons of cross sectional surveys either using student [6] or population based samples [14]. In these circumstances it is not clear that the samples are effectively comparable.

Mothers in this study are primarily of the Baby Boomer generation (born 1946–63), with a small number early Generation X (born 1964–81). These are women who were reared in the context of the protest movements common in 1970s generation and the threat of a potentially catastrophic nuclear war. Women in this generation joined the paid workforce in increasing numbers as their children left home. This generation lived a relatively stable lifestyle coming from families where marriages rarely led to divorce, but were the first generation to divorce their partners in substantial numbers [22].

Daughters in this study were of the millennial generation, known as generation Y (born 1977–2001). In Australia this generation experienced long periods of almost uninterrupted economic prosperity. Historically this is the most educated generation of women ever. Compared to previous generations, they have delayed marriage and having children and more frequently adopt single marital status and single parenthood as a life choice. Most of the women in this generation are in the workforce and aspire to achievement [23].

In this study we aim to test the proposition that the different life courses experienced by the mothers and daughters in this study may have contributed to differences in their life histories of mental illness. We compare mothers and their daughters across four lifetime ever diagnoses: major depressive disorder, generalised anxiety disorder, panic disorder and post-traumatic stress disorder (PTSD). We hypothesise that there is a generational change in the rates of mental illness with daughters having higher rates of mental disorders than their mothers.

## Methods

### Study design

Data are from the Mater-University of Queensland Study of Pregnancy (MUSP) and its outcomes. The details of the study methodology appear elsewhere [24, 25]. Briefly, 6753 mothers giving birth to 7223 children over the period 1981–1983 were recruited on the basis they were consecutive women attending an antenatal clinical service for their first obstetric assessment at a major public maternity hospital in Brisbane, Australia.

**Fig. 1:**
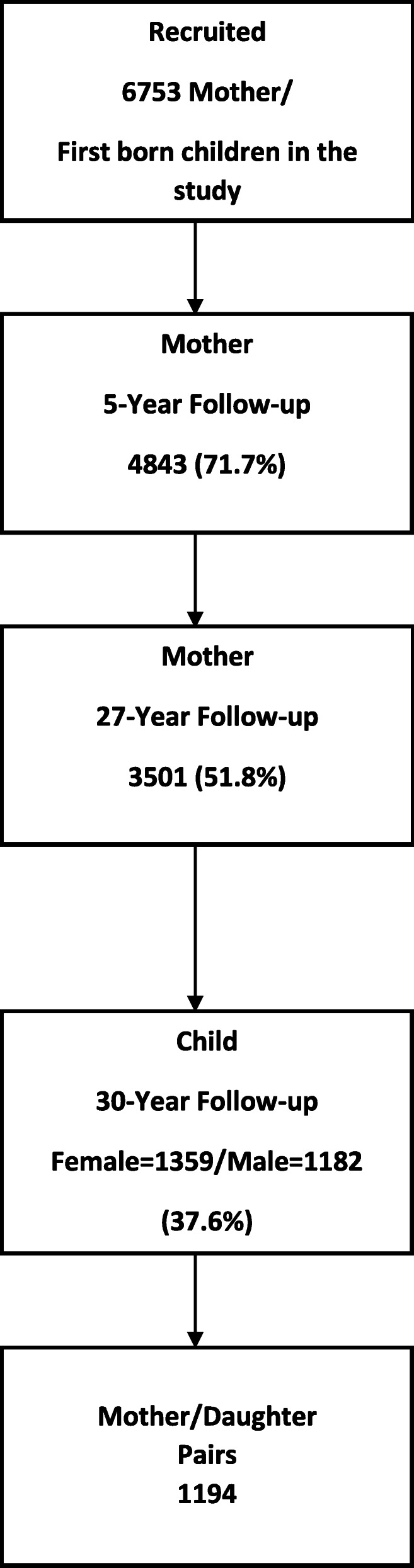
Sample Retention to 30 Years. 6753 pregnant women were recruited into the study at their first obstetrical visit (1981–3). These women and their children were followed-up five years after the birth (1986–88), and the mothers were also follow-up 27 years after the birth (2008–11) and the offspring, 30 years after the birth (2010–14). The CIDI was administered to mothers at the 27-year follow-up and to daughters at the 30-year follow-up

Figure 1 provides a flow chart which documents sample attrition. After the 14-year follow-up, mothers and children were treated as separate samples.

Mothers recruited to the study in 1981–3 were administered the Composite International Diagnostic Interview (CIDI) at the 27 year follow-up when they were a mean age of 53.0 years (SD = 5.0). Interviews were administered either face-to-face or over the phone. Some 3501 mothers (excluding those women who had repeated pregnancies with two offspring recruited) completed the CIDI at the 27 year follow-up. Their offspring were administered the CIDI at the 30 year follow-up (2010–2014) when their mean age was 30.4 (SD = 1.1) years. In total 2541 offspring provided useable data for the CIDI at the 30 year follow-up, of whom 1359 were females. We restrict comparisons to mother-daughter pairs for whom CIDI data are available (*N* = 1194).

### Composite international diagnostic interview (CIDI-auto)

The CIDI is a structured clinical interview using criteria from the Diagnosis and Statistical Manual of Mental Disorders (4th edition) (DSM-IV) and International Classification of Diseases (ICD) to determine the diagnostic status of respondents [26]. The CIDI is the most widely used of the available measures to estimate population rates of DSM/ICD mental illnesses.

The CIDI has good test-retest reliability and good agreement with clinical diagnoses for a number of diagnostic categories [27]. Early validation used a criterion (gold standard) of two clinicians who agreed on a diagnosis then compared this with CIDI-Auto diagnoses [28]. The sensitivity and specificity of the CIDI-Auto varied by diagnostic category with major depression and panic disorder having good sensitivity and moderate specificity. Generalised anxiety disorder had poor sensitivity but moderate specificity. A larger scale primary health care sample has compared CIDI diagnoses with the WHO semi-structured SCAN interview [29]. This study finds good sensitivity and specificity (but poorer concordance) for depression, panic disorder and generalised anxiety disorder. A similar study using population data and the Structured Clinical Interview for DSM-IV as the criterion reports moderate sensitivity and high specificity for a wide number of anxiety disorders and major depressive disorder [30]. The modest level of reliability associated with CIDI-Auto diagnoses may reflect the nature of psychiatric diagnoses (the state of the science) more than the use of the CIDI. Thus studies of diagnostic reliability using both forensic samples [31, 32] and psychiatric patient samples [33] point to reliability estimates (test-retest) which are of the same order suggested when CIDI-Auto diagnoses are assessed against clinical interviews. Studies using specifically briefed and trained interviewers [28] provide good supportive evidence for the validity and reliability of the CIDI-Auto and modest levels of sensitivity and specificity suggested by other studies need to be interpreted in the context that clinical diagnoses in population settings are affected by similar limitations.

### Age of onset of CIDI disorder (AOO)

Mothers and their daughters were assessed for their lifetime ever mental disorders. In order to make this as valid a comparison as possible, the mother’s mental disorders were restricted to reports when the age of onset of a life time ever disorder was before 30 years.

Early versions of the CIDI involved the collection of age of onset data that often proved inaccurate [34]. Subsequent versions of the CIDI included additional questions intended to locate the symptoms in the context of blocks in the life course such as attendance at primary school, high school, university and employment. There was less concern with exact dating than with determining the life course context within which the first episode was observed. Much of what is known about the accuracy and reliability of age of onset recall is derived from studies of persons who have experienced depression [35–39]. Very little is known about the reliability of age of onset data for conditions other than depression.

This study has an inherent risk of recall bias [35, 37] with mothers interviewed in their later adult life more likely to fail to recall an episode of illness which occurred before they were 30 years compared to their daughters for whom the episode of illness is more recent. There is consistent evidence that more severe episodes produce better recall [34, 36, 39]. To reduce this risk of recall bias, we have chosen the duration of an episode of illness as a proxy marker for severity. In an additional sensitivity analysis we have recalculated daughter-mother differences under varying assumptions about the extent to which mothers may be more subject to not recalling a past episode.

### Confounders

Confounders were selected on the basis that they have been previously implicated in predicting mental illness and were available in both the mother and daughter data set. Four measures of the mother’s and daughter’s sociodemographic characteristics were used in his study. The mother’s employment status, occupational standing or social class and marital status were each taken from the 5 year follow-up. At this follow-up mother’s mean age is most similar to the daughter’s at the time the daughter’s CIDI data are obtained. Mother’s education was taken from the recruitment visit with mothers generally not adding to their education levels after the birth of their child. For the daughter details of her employment status, education level, occupational class and marital status are all taken from self-reports provided at the 30 year follow-up.

### Statistical analysis

Initial analysis comprised a test of the association between the sociodemographic characteristics of daughters and mothers as these are related to four DSM-IV diagnoses (two tailed chi squared). To assess generational changes in mental illness we compare the lifetime ever rates of four DSM-IV diagnostic groups; major depression, generalised anxiety disorder, panic disorder and PTSD by daughters and the mothers. We use McNemars test of marginal homogeneity to compare the crude rates and then adjusted the McNemars test for a number of daughter and maternal sociodemographic characteristics. We compute the adjusted McNemars marginal odds ratio with 95% confidence intervals. This adjustment is to address the possibility that socio-demographic changes affecting the day-to-day lives of women may account for generational changes in anxiety and depression. We then repeat these analyses stratifying for duration of episode of illness. All these analyses are undertaken using STATA 14.1.

Additional analyses are undertaken using SPSS 25 and Medcalc online odds ratio calculator. Episodes of illness were stratified into those that were persistent (≥12 months) and less persistent (< 12 months) on the basis that persistent episodes of illness are more likely to be recalled by the mothers and the comparisons with their daughters are less prone to recall bias. For these additional analyses we address the possibility that mothers have “forgotten” 10, 20 and 30% of both more and less persistent diagnosed conditions. These additional tables are interpreted as sensitivity analyses intended to identify a possible level of recall bias which would compromise the study findings.

## Results

Table 1 compares key sociodemographic characteristics of mothers and daughters. With the exception of education, mothers’ sociodemographic characteristics are taken from the 5 year follow-up when mothers were a mean age of about 30 years. Mothers described themselves primarily as housewives, with a partial or complete secondary education, working overwhelmingly in clerical/service/sales positions (when employed) and of married status. Their daughters at 30 years are generally employed, the majority with a tertiary education, working either in clerical/service/sales or in professional and managerial positions. By 30 years of age 83.1% of mothers and 49.7% of daughters were married.

**Table 1:** Sociodemographic Characteristics of mothers (1981–4) and their daughters (2010–14)

	Mother %	Daughter %	X^**2**^, ***p***-value
**Employment**			
Other (Home Duties)	48.6 (529)	23.0 (249)	
Unemployed	9.6 (104)	5.4 (58)	
Employed	41.8 (455)	71.6 (775)	**197.1,** ***p*** **< .001**
**Education**			
Secondary Only	77.1 (912)	31.2 (338)	
Other Tertiary	19.5 (231)	35.6 (385)	
University	3.4 (40)	33.2 (359)	**553.7,** ***p*** **< .001**
**Occupational Class**			
Manual/Trades/Other	23.9 (252)	6.7 (73)	
Clerical/Service/Sales	69.3 (732)	54.0 (586)	
Professional/Managerial	6.8 (72)	39.3 (426)	**366.1,** ***p*** **< .001**
**Marital Status**			
Single	3.4 (37)	19.8 (215)	
Living Together	5.2 (56)	24.4 (264)	
Separated/Widowed/Divorced	8.3 (90)	6.1 (66)	
Married	83.1 (903)	49.7 (539)	**356.5,** ***p*** **< .001**

For daughters there are a strong and consistent set of associations between their sociodemographic characteristics and their lifetime ever experiences of most CIDI diagnoses (Table 2). Unemployed daughters have higher rates of major depression, generalised anxiety disorder, panic disorder, and PTSD. Daughters working manual occupations have higher lifetime ever rates of major depression, generalised anxiety disorder and PTSD. Daughters who are separated/widowed/divorced have higher rates of major depression.

**Table 2:** Daughter sociodemographic characteristics associated with lifetime ever CIDI diagnoses (percent lifetime ever diagnosis)

	Major Depression (MDD)	Generalised Anxiety Disorder	Panic Disorder	PTSD
**Employment (30 Yr F/U)**				
Other – Home Duties (*N* = 249)	25.3%	7.3%	2.8%	15.0%
Unemployed (*N* = 58)	46.6%	19.3%	10.3%	33.9%
Employed (*N* = 775)	24.8%	7.5%	4.5%	10.0%
	**X**^**2**^ **= 13.4,** ***p*** **< .001**	**X**^**2**^ **= 10.3,** ***p*** **< .01**	**X**^**2**^ **= 6.3,** ***p*** **= .04**	**X**^**2**^ **= 29.8,** ***p*** **< .001**
**Education (30 Yr F/U)**				
Secondary Only (*N* = 338)	27.8%	9.2%	4.4%	16.1%
Other Tertiary (*N* = 385)	25.5%	9.1%	4.9%	13.9%
University (*N* = 359)	25.1%	6.1%	3.9%	7.2%
	X^2^ = 0.8, *p* = ns	X^2^ = 2.9, *p* = ns	X^2^ = 0.5, *p* = ns	X^2^ = 13.9, *p* < .001
**Occupational Class (30 Yr F/U)**				
Manual/Trades/Other (*N* = 73)	41.1%	13.7%	9.6%	25.7%
Clerical/Service/Sales (*N* = 586)	25.3%	9.6%	4.3%	13.2%
Professional/Managerial (*N* = 426)	24.9%	5.2%	3.8%	9.2%
	**X**^**2**^ **= 9.0,** ***p*** **= .01**	**X**^**2**^ **= 9.7,** ***p*** **< .01**	X^2^ = 5.1, *p* = .08	**X**^**2**^ **= 15.8,** ***p*** **< .001**
**Marital Status (30 Yr F/U)**				
Single (*N* = 215)	33.5%	8.4%	5.1%	11.7%
Living Together (*N* = 264)	23.5%	9.2%	6.8%	13.2%
Separated/Widowed/Divorced (*N* = 66)	42.4%	15.2%	4.5%	19.7%
Married (*N* = 539)	22.3%	6.5%	3.0%	11.3%
	**X**^**2**^ **= 20.3,** ***p*** **= <.001**	X^2^ = 6.7, *p* = .08	X^2^ = 6.5, *p* = ns	X^2^ = 4.1, *p* = ns

For mothers the associations between their sociodemographic characteristics and DSM-IV lifetime ever diagnoses appear to be neither strong nor consistent (Table 3). Employed mothers have higher rates of lifetime ever generalised anxiety disorder, and unemployed mothers have higher rates of PTSD. Separated/widowed/divorced mothers have higher rates of lifetime ever major depression and PTSD. Women who are married have lower rates of major depression and PTSD.

**Table 3:** Mother sociodemographic characteristics associated with lifetime ever CIDI diagnoses (percent lifetime ever diagnosis)

	Major Depression (MDD)	Generalised Anxiety Disorder	Panic Disorder	PTSD
**Employment (5 Yr F/U)**				
Other (*N* = 529)	20.4%	7.8%	4.5%	13.8%
Unemployed (*N* = 104)	28.8%	9.6%	5.8%	24.0%
Employed (*N* = 455)	23.5%	13.2%	4.8%	14.7%
	X^2^ = 4.0, *p* = ns	**X**^**2**^ **= 7.9,** ***p*** **= .02**	X^2^ = 0.3, *p* = ns	**X**^**2**^ **= 7.2,** ***p*** **= .03**
**Education (Recruitment)**				
Secondary Only (*N* = 912)	22.8%	10.4%	5.3%	16.8%
Other Tertiary (*N* = 231)	24.2%	12.1%	3.9%	13.4%
University (*N* = 40)	22.5%	7.5%	5.0%	7.5%
	X^2^ = 0.2, *p* = ns	X^2^ = 1.0, *p* = ns	X^2^ = 0.7, *p* = ns	X^2^ = 3.7, *p* = ns
**Occupational Class (5 Yr F/U)**				
Manual/Trades/Other (*N* = 252)	23.0%	10.7%	3.6%	16.7%
Clerical/Service/Sales (*N* = 732)	22.1%	10.5%	5.2%	15.2%
Professional/Managerial (*N* = 72)	20.8%	5.6%	2.8%	6.9%
	X^2^ = 0.2, *p* = ns	X^2^ = 1.8, *p* = ns	X^2^ = 1.7, *p* = ns	X^2^ = 4.2, *p* = ns
**Marital Status (5 Yr F/U)**				
Single (*N* = 37)	21.6%	8.1%	8.1%	21.6%
Living Together (*N* = 56)	23.2%	10.7%	5.4%	23.2%
Separated/Widowed/Divorced (*N* = 90)	35.6%	17.8%	6.7%	32.2%
Married (*N* = 903)	21.0%	9.4%	4.4%	12.9%
	**X**^**2**^ **= 10.0,** ***p*** **= .02**	X^2^ = 6.5, *p* = ns	X^2^ = 1.9, *p* = ns	**X**^**2**^ **= 27.9,** ***p*** **< .001**

In Table 4 we compare daughter-mother dyads and have only considered mothers’ diagnostic events if the age-of-onset was 30 years or less. Additional file 1 provides details of the age-of-onset excluded cases with about 50% of most mother age-of-onsets occurring after 30 years of age.

**Table 4:** Comparison of CIDI diagnoses (lifetime ever) for mothers (first pregnancy) and their daughters (only for mother and daughters where diagnostic data is available for both)*

		Daughter	Mother	D/M Odds Ratio (95% CI)Unadjusted	D/M** Odds Ratio (95% CI)Adjusted***
		N%	N%		
Major Depressive Disorder	Yes	26.5	11.0	**2.89(2.27, 3.69)**	**2.91(2.28,3.72)**
	No	73.5	89.0		
		1194	1194		
Generalised Anxiety Disorder	Yes	8.2	4.6	**1.91(1.29,2.83)**	**1.92(1.30,2.86)**
	No	91.8	95.4		
		1188	1188		
Panic Disorder	Yes	4.4	2.5	**1.94(1.14,3.32)**	**1.95(1.14,3.34)**
	No	95.6	97.5		
		1194	1194		
PTSD	Yes	13.1	10.3	1.29(0.97,1.71)	1.29(0.97,1.72)
	No	86.9	89.7		
		1178	1178		

*McNemar’s test for marginal homogeneity

**Adjusted for mother’s employment and education, and daughter’s employment, education, occupational class and marital status ( N = 972)

***Numbers vary slightly with missing values for some diagnostic categories

Comparing daughter-mother pairs, daughters have higher rates of lifetime ever major depression, generalised anxiety disorder and panic disorder. Daughters appear to experience almost three times the rate of major depression compared to their mothers, and almost twice the rate of generalised anxiety disorder and panic disorder. Adjustment for daughter and mother sociodemographic characteristics appears to have little effect on these findings. We have repeated this analysis in Additional file 1 (mothers and female children but without pairing mothers and daughters). The findings are generally similar to those observed in Table 4.

The sample was then stratified by illness duration. Comparing daughters and mothers with age-of-onset 30 years or less we note that the data are very similar suggesting there has been little or no change between generations in the age-of-onset of their disorders (Additional file 1). Considering persistent disorders (12-months or longer), generalised anxiety disorders and panic disorder, daughters have higher rates than do their mothers (Table 5). For persistent disorders there was no difference between the generations in rates of major depression and PTSD.

**Table. 5:** Comparing daughters and mothers for persistent disorders, first episode only (DSM-IV). Odds Ratio (95% CI) daughter/mother with inflation for mother recall

		Persistent Condition^a^					Less Persistent Condition^b^				
		Daughter		Mother		D/M Odds Ratio (95% CI)	Daughter		Mother		D/M Odds Ratio (95% CI)
		N	%	N	%		N	%	N	%	
**Major Depressive Disorder**	No	1126	94.5	1141	95.8	1.32(0.91,1.91)	839	65.4	1005	93.1	**3.85(2.96,5.01)**
	Yes	65	5.5	50	4.2		241	22.3	75	6.9	
**Generalised Anxiety Disorder**	No	1122	95.2	1151	97.7	**2.13(1.32,3.42)**	1056	96.4	1076	98.3	**2.09(1.19,3.67)**
	Yes	56	4.8	27	2.3		39	3.6	19	1.7	
**Panic Disorder**	No	1147	96.1	1164	97.6	**1.61(1.01,2.57)**	1114	99.5	1119	99.9	6.03(0.72,50.27)
	Yes	46	3.9	29	2.4		6	0.5	1	0.1	
**PTSD**	No	1058	89.8	1075	91.3	1.18(0.90,1.56)	933	96.7	951	98.5	**2.33(1.24,4.36)**
	Yes	120	10.2	103	8.7		32	3.3	14	1.5	
**Sensitivity Analysis**		Mother Episodes 10% Inflation		Mother Episodes 20% Inflation		Mother Episodes 30% Inflation	Mother Episodes 10% Inflation		Mother Episodes 20% Inflation		Mother Episodes 30% Inflation
Major Depressive Disorder		1.19(0.82,172)		1.00(0.76,1.56)		1.00(0.70,1.42)	**3.45(2.65,4.50)**		**3.16(2.44,4.09)**		**2.88(2.24,3.70)**
Gen Anxiety Disorder		**1.91(1.23,3.00)**		**1.79(1.15,2.78)**		**1.63(1.06,2.51)**	**1.89(1.10,3.23)**		**1.72(1.02,2.90)**		1.58(0.95,2.63)
Panic Disorder		1.46(0.92,2.30)		1.32(0.84,2.06)		1.21(0.78,1.87)	–		–		–
PTSD		1.07(0.82,1.40)		0.96(0.74,1.26)		0.88(0.68,1.15)	**2.17(1.17,4.04)**		**1.91(1.05,3.47)**		**1.80(1.01,3.24)**

^a^Persistent = 12 months or longer

^b^less persistent = less than 12 months

For less persistent disorders, daughters report substantially higher rates than their mothers for major depression, generalised anxiety and PTSD (almost all panic disorders are of persistent duration). These shorter duration episodes are arguably more likely to be subject to recall bias.

In Table 5 we further test the possibility that poorer maternal recall of past episodes may account for our findings. For both persistent and less persistent conditions we inflate maternal reports (cases) by 10, 20 and 30% and recalculate daughter-mother ratios of DSM-IV mental disorder. For the persistent disorders only Generalised Anxiety Disorder remains significantly higher for daughters compared to their mothers. For the less persistent disorders inflating maternal reports even by 30% does not substantially alter the finding that daughters report substantially higher levels of less persistent symptoms and disorders than do their mothers.

## Discussion

Despite some evidence that rates of mental illness in more recent cohorts have greatly increased [6, 12] and that rates of treatment for mental illnesses have been rapidly escalating [18–20], doubts remain about the extent to which reported changes may reflect a greater willingness to report symptoms, an increased sensitivity to symptoms and/or the increased availability of treatment services [40]. There is also some evidence that rates of depression may have increased over time, particularly for younger women [41].

In the current study we have restricted our comparisons to a mother-daughter dyad and used a structured clinical diagnostic assessment based upon DSM-IV criteria, to determine whether there has been a generational change in four categories of mental illness.

Comparing mother-daughter dyads we find, as anticipated, that the daughters are mostly employed or otherwise in the workforce (a minority of mothers were employed), much more likely to have a university level education and much more often employed as professionals or managers. Some 46.6% of unemployed daughters meet the CIDI criteria for a lifetime ever major depression compared to 28.8% of unemployed mothers who report ever experiencing depression. Of the daughters who are employed in manual/trades groups, 41.1% report ever experiencing major depression compared to 23.0% of their mothers in this occupational category. The pattern of results suggests that the employment and occupational circumstances of daughters are more strongly related to their mental health than is the case for their mothers. In interpreting this finding, we speculate that the mental health of daughters may be more closely tied to their employment and workforce participation than was the case for their mothers. While there are marital status differences comparing mothers and daughters, the marital status of women with the highest rates of mental illness are those who are separated/widowed or divorced. For this category of marital status there appear to be (slightly) fewer daughters (6.1%) compared to mothers (8.3%). While there are changes in the characteristics of mothers and daughters than might lead to decreased levels of mental illness (fewer unemployed daughters than mothers, fewer separated/widowed/divorced daughters than mothers) there may also be changes that might lead to increased levels of mental illness (more daughters are single compared to their mothers). Overall our findings do not point to social and demographic changes in the life circumstances of mothers and their daughters that are likely to have a substantial impact when comparing the mental health of these two generations of women.

The direct comparison of the lifetime ever experiences of mental illnesses confirms that adjustment for the changes in the life circumstances of these two generations of women has no impact on the estimates of the extent to which daughters experience substantially higher levels of lifetime ever major depressive disorder, generalised anxiety disorder and panic disorder. However disaggregating the comparison groups into disorders of greater and lesser persistence, and further inflating maternal rates to provide for the lower levels of recall of past episodes, a possible consequence of the mothers’ older age at interview, suggests that higher rates of major depression and PTSD are of less persistent conditions. For generalised anxiety disorders somewhat higher rates are observed for daughters for both persistent and less persistent episodes.

### Limitations

The findings should be interpreted in the context of some limitations of the study. Recruitment was of consecutive pregnant women and our findings may not be generalised to male parents and their male offspring. Further, the sample is selected in a developed western economic system and the findings are not generalisable to middle- and lower-income countries.

A major concern is that mothers were substantially older than their daughters when they completed the CIDI. To address this concern we have only included mothers’ diagnoses if these had an age of onset of 30 years or less. Further we have distinguished more and less persistent episodes, the latter arguably more subject to recall bias. We have also undertaken sensitivity analyses involving adjustment for possible rates of maternal failure to recall past episodes.

In comparing daughter and mother’s recall of age-of-onset of four disorders, some additional caveats need to be considered. The natural histories of the four disorders differ and they may consequently be subject to different levels of recall bias. Major depressive disorder appears to involve a repeated combination of chronic (persistent) and acute episodes [42]. By contrast, PTSD involves exposure to a stressful and/or traumatic event, the impact of which may diminish for many over time [43, 44]. Although both daughters and mothers, in the CIDI, are being asked to recall symptoms which they may have experienced in the past, the extent to which their memories are affected by the period of recall is unknown. In one of the only studies which assesses the reliability of longer term recall, a 25-year follow-up of clinically diagnosed (and treated) persons with depression, some 70% recalled having experienced some symptoms of depression while about 50% recalled sufficient symptoms to meet the clinical criteria for depression [42]. In this context a sensitivity analysis with maternal recall (compared to daughter recall) inflated by 10 to 30% appears appropriate.

Nevertheless it is possible that recall bias impacts on episodes of longer durations as well leaving the possibility of recall bias as an explanation of our findings unresolved. While our study has the strength of using a structured diagnostic interview administered to both daughters and mothers, there remain concerns about the fidelity of our comparison of the level of mental illness experienced by daughters and mothers. In the context of significant community and academic interest in generational changes in rates of mental illness, there remain difficulties in comparing generations that previous researchers have also been unable to resolve. Further, had the comparison included younger or older women – it is possible that the findings would be different. Our findings are only relevant to the mental health of younger women.

## Conclusions

We find that daughters are much more likely to report a wide range of less persistent mood disorders than did their mothers. Overall we conclude that the more recent generation of women only experience somewhat higher rates of persistent anxiety disorders. The most recent generation of women in this study also appear to experience higher rates of a number of more transient mood and anxiety disorders.

Three key issues arise from our findings. The first concerns whether the observed increase in mental disorders experienced by daughters might be attributed to underlying societal changes. Were this the case and given that societal changes are likely to be ongoing, treatment for mental illness might be characterised as a strategy for mitigating the harmful consequence of societal change. In the event our findings do not point to societal changes that may have contributed to the increase in episodes of mental illness. Second, if our findings are correct then the increase in mental illnesses over a generation is primarily in shorter term conditions which appear to remit over time. There is a need to consider the wisdom and value of increasing treatment services for conditions which are more transient and remitting. Third, we have noted the rapid increase in the use of treatment services by those with a mental illness. It is important to consider whether treatments for mental illnesses of shorter duration have different benefits than treatments provided to those with more chronic conditions.

## Supplementary Information

**Additional file 1: Appendix A.** CIDI (DSM-IV) Diagnoses for Mothers. **Appendix B.** Comparison of CIDI Diagnoses (Lifetime Ever) for Mothers (First Pregnancy) and their Daughters (only Mothers of Daughters included). **Appendix C.** Comparing Daughters and Mothers, Age of Onset of Selected DSM-IV Disorders (Mean & 95% CI).

## Data Availability

The datasets used and/or analysed during the current study are available from the corresponding author on reasonable request.

## Abbreviations

DSM-IV: Diagnosis and Statistical Manual of Mental Disorders 4th Edition
CIDI: Composite International Diagnostic Interview
PTSD: Post-traumatic stress disorder
MUSP: Mater-University of Queensland Study of Pregnancy
ICD: International Classification of Diseases
WHO-SCAN: World Health Organisation Schedules for clinical assessment in neuropsychiatry
AOO: Age of onset (of CIDI disorder)
STATA: Software for statistics and data science
SPSS: Statistical Package for the Social Sciences
