# Supplementary material for: Does the millennial generation of women experience more mental illness than their mothers?
Source: BMC Psychiatry. 2021 Jul 17;21:359. doi: 10.1186/s12888-021-03361-5 (PMC8285825; doi:10.1186/s12888-021-03361-5)
Supplement: Supplementary file 1 — Additional file 1: Appendix A. CIDI (DSM-IV) Diagnoses for Mothers. Appendix B. Comparison of CIDI Diagnoses (Lifetime Ever) for Mothers (First Pregnancy) and their Daughters (only Mothers of Daughters included). Appendix C. Comparing Daughters and Mothers, Age of Onset of Selected DSM-IV Disorders (Mean & 95% CI). [file 12888_2021_3361_MOESM1_ESM.docx]

Appendix A: CIDI (DSM-IV) Diagnoses for Mothers

(N=1194 Mothers)

|  | ≤30 Yrs % | >30 Yrs % | Total N |
| --- | --- | --- | --- |
| Major Depression (MDD) | 47.6 | 52.4 | 275 |
| Generalised Anxiety Disorder | 44.0 | 56.0 | 125 |
| Panic Disorder | 50.0 | 50.0 | 60 |
| PTSD | 66.5 | 33.5 | 185 |

Appendix B: Comparison of CIDI Diagnoses (Lifetime Ever) for Mothers (First Pregnancy) and their Daughters (only Mothers of Daughters included).

|  |  | Daughter | | Mother | | D/M Relative Risk*  (95% CI) |
| --- | --- | --- | --- | --- | --- | --- |
|  |  | N | % | N | % |  |
| Major Depressive Disorder | Yes | 366 | 26.9 | 218 | 12.7 |  |
|  | No | 993 | 73.1 | 1504 | 87.3 | **2.13(1.83,2.48)** |
|  |  | 1359 |  | 1722 |  |  |
| Generalised Anxiety Disorder | Yes | 112 | 8.3 | 87 | 5.1 |  |
|  | No | 1242 | 91.7 | 1633 | 94.9 | **1.64(1.25,2.14)** |
|  |  | 1354 |  | 1720 |  |  |
| Panic Disorder | Yes | 59 | 4.3 | 42 | 2.4 |  |
|  | No | 1300 | 95.7 | 1680 | 97.6 | **1.78(1.21,2.63)** |
|  |  | 1359 |  | 1722 |  |  |
| PTSD | Yes | 182 | 13.5 | 185 | 10.8 |  |
|  | No | 1164 | 86.5 | 1531 | 89.2 | **1.25(1.04,1.52)** |
|  |  | 1346 |  | 1716 |  |  |

*Medcalc

Appendix C: Comparing Daughters and Mothers, Age of Onset of Selected DSM-IV Disorders (Mean & 95% CI)

|  | Daughter | Mother | |
| --- | --- | --- | --- |
|  |  | ≤ 30 Yrs | All Mothers |
| Major Depressive Disorder | 19.3(18.7,19.9) | 19.8(19.1,20.5) | 30.9(30.1,31.8) |
| Generalised Anxiety Disorder | 18.4(17.0,19.7) | 18.4(17.2,19.6) | 31.4(30.0,32.8) |
| Panic Disorder | 19.2(17.5, 21.0) | 18.8(17.3,20.3) | 29.0(27.2,30.8) |
| PTSD | 17.7(16.6,18.8) | 15.2(14.5,16.0) | 25.2(24.0,26.4) |
